# The Cure of Pulmonary Tuberculosis upon the Principles of Nutrition

**Published:** 1892-03

**Authors:** Karl Von Ruck

**Affiliations:** Asheville, N. C.


### THE CURE OF PULMONARY TUBERCULOSIS UPON THE PRINCIPLES OF NUTRITION.

BY KARL VON RUCK, B. S., M. D., ASHEVILLE, N. C.

(Concluded.)

Perfect general nutrition, in the sense I employ the word, means the harmonious and normal functional activity of the living organism, and when so much lung has been hopelessly destroyed that an efficient respiratory function is no longer possible, or if other important organs have become, or are previously so damaged that a sufficient degree of nutritive and functional activity is lost, the case has of necessity become incurable.

Constitutional and previous disease may have left everlasting impressions, even the partial removal of which, would be entirely impossible, and for this reason some victims of tuberculosis are doomed to succumb with the moment of infection, and nothing short of its prevention can be of avail to them.

On the other hand, there are perhaps exceptional cases which, under a better constitution, with intact digestive and assimilative powers, have overcome the early manifestations of the disease contracted under a temporary predisposition with little or no aid on our part, and others who might possibly have recovered under less favorable conditions than we believed essential for their welfare. As to those, we need never regret having done our whole duty, but we need to concern ourselves much more seriously with the great majority who die of the disease for want of proper treatment and management at a time when they are in the best condition to derive benefit, and the partial results which we still accomplish in the advanced stages admonish us constantly, that an earlier systematic effort would probably have led to a complete recovery.

Let, therefore, no physician who advises tubercular patients, be satisfied with partial efforts and put his trust in the early stage of the disease, expecting that with little or no aid, nature alone will effect a cure.

Let no one hope to overcome even this stage with a drug prescription or any mode of administration of drug remedies, even if they have the recommendation of specifics and come indorsed by high authorities.

Every patient who has reached an advanced stage and finally dies, was at one time in this early stage where he might perhaps have recovered.

The physician who thinks he has done his duty, even when he has ordered a change of climate, and tells his patient that such a change is all that is essential for his recovery, knows little of the cure of pulmonary tuberculosis. Let him look back over his past experience and enquire what has become of all the tubercular patients to whom he gave prescriptions for hypophosphites, creasote, cod-liver oil and for other drugs, or whom he sent upon a journey for health on a tour of the climatic resorts, first to one place, then to another, the patient at such places being set adrift like a vessel without captain or pilot, and let him realize how few, if any, have made a recovery.

I have done this with 243 cases which I advised and treated upon a combination of such plans in private practice, and of all that number, only 15 were still alive in from two to eight years after they came under my care.* Ten of these were considered to have recovered—a poor chance indeed for the individual case.

I have already stated that every remedy—be that a drug, a reputed specific, a change of climate, a dietetic, or hygienic measure of whatever nature—must be shown to favorably influence the nutritive processes, either local or general, or both, of the case under consideration, to entitle it to our con-

* "The Management of Pulmonary Phthisis." *Therap. Gazette.* Aug, 15 1889.

fidence and recommendation, and it goes without saying that on the other hand every remedy which is indifferent or acts injuriously, or any conduct of the patient which is detrimental directly or indirectly to the maintaining and increasing of his nutritive processes, we must avoid and interdict.

Perhaps most important among all our resources, is the employment of climate; and the influence it exerts upon the course of tuberculosis need not be sought in some peculiar hidden or mysterious agency which resides, for instance, in Asheville or Colorado Spring, nor in any particular attribute or prominent meteorological feature of a particular climatic resort.

Why, for instance, should we insist that all the benefit climate can bestow must come from a dry atmosphere, and the drier it is the better? If it were so, we could perhaps produce it artificially at home; and although Asheville, N. C., the field of my present labors, has also a just claim to a relatively dry climate, I am not blind to the fact that at certain seasons of the year certain classes of patients do well—yes, as well as anywhere, at places where the air contains much more moisture than the dry air advocates would find sufficient to preclude all chances for improvement. Climate presents such complex conditions and so many factors enter into it, that we cannot narrow its influence down to one single feature of it.

If we look at climatic influences as affecting nutrition, the mystery disappears and we can see how a locality where there is a preponderance of sunshine, in the absence of extreme conditions of temperature and strong winds, with fine scenery, having a pure relatively dry air, free from dust and irritants, allowing of much out-of-door life, with sufficient elevation to favor a better circulation, would allow of better nutritive processes than one where the reverse conditions obtain.

Such a continuous influence has been found very valuable, and my observations upon patients justify the opinion that unless in the presence of active disease or complications, when the patient is under otherwise favorable conditions and proper management, the nutritive functions revive and improve soon after arrival from a less favorable locality.

The otherwise favorable conditions and proper management are, however, so important that their absence can not only negative all the benefits the climate can bestow, but favor relapses and advances of the disease. The *proper* use of the climate is equally essential, and the amount of out-of-door life, especially the allowable exercise, needs to be regulated and adapted to the conditions of the individual patient, at the time being.

Patients at climatic resorts require care and comforts, and require properly-heated, well-ventilated houses; must have a diet adapted to the conditions of their digestive organs and powers of assimilation, and of the best quality; they must avoid the injurious influences of over-exertion, exposure and other indiscretions, the same as they should at home, *climate furnishing no protection against follies, indiscretions and errors, whether from want of proper knowledge or sufficient self-control.*

Painstaking professional advice based upon the present condition of the patient, admonitions and control, as well as approval and encouragement, special treatment both for the disease itself which can aid, and for other concomitant or intercurrent affections which hinder the best nutritive processes, as for instance, the removal of nasal obstruction to freer respiration, of catarrhal affections of the upper air-passages and of the digestive organs, etc., etc.,—are as essential at the climatic resorts as they are without the advantage of climate, if we desire to keep our patient at his best and clear the way of obstacles to an uninterrupted favorable progress.

The facts are that consumptive patients require an ever-present vigilance on the part of their medical adviser, not only for the removal of the many hindrances to the best nutrition, but for the purpose of preventing their occurrence, and thereby the relapses from the favorable progress already made. One single slip from the ideal course may lose all that has been gained by months of improvement. They also require some systematic observations of their general condition, as well as of the local process in the lung; and frequent records and comparisons by which we can judge of the progress of the case are essential, that we may be able to give the best advice. Every detail of the patient's life must be so arranged and controlled that it will lead to the nearest approach possible to a perfect functional activity of the organism, and nothing must be too trivial for the managing physician to give his attention to which can be turned to the advantage of the patient. Such management is not likely to be carried out, except in a special institution.

If the patient can have such conditions at home and has to do without them at a climatic resort, I am convinced that he had better take his chances without the advantages of climate.

Let any physician, who cares to try, take an early-stage case of pulmonary tuberculosis in his own house and under his personal control and constant observation; let him carry out painstakingly and persistently the method of management here only indicated; let him bear in mind the principles of nutrition as the object of his endeavors constantly, and weigh every advice or interdiction he gives and every means he employs in the light of its favorable or unfavorable influence upon the local or general nutritive processes, and he will find himself not so helpless in his endeavors as he might believe himself to be, before making the experiment.

Let him also take advantage of the experience of such as have so cared for many cases until his own experience can guide him in all matters, and he will find that he can aid nature sufficiently to crown his labors with considerable success, and this in almost any climate of this country. If the patient can have the important aid of a favorable climate in addition, so much the better.

I have myself labored in this manner in an indifferent climate, and the results obtained are still permanent in over 40 per cent. of the 58 cases treated after from three to six years since their discharge. The majority of these cases were, however, in the early stage.*

The diet in pulmonary tuberculosis I have not yet spoken of, and it would seem strange should I not specially mention it, since I base the success of treatment upon the degree of nutrition in the particular case.

I have heretofore published my views in a paper entitled

* "The Management of Pulmonary Phthisis," *Therapeutic Gazette*, Aug. 15, 1889.

"Some Observations upon the Gastro-Intestinal Disorders and the Diet in Pulmonary Phthisis,"* and have shown there that a proper diet for the best nutrition is, of course, of greatest importance.

There is, however, according to my experience, no possibility of laying down absolute rules as to what and how much all patients shall eat and drink, and constant individualization is here necessary too, as it is in all our therapeutic endeavors.

One of the greatest secrets of success is the prevention and removal of the various digestive disturbances. In their absence, and also in the absence of fever, I allow my patients a liberal choice from the best of a general dietary, interdicting only such articles as may reasonably be feared to disagree or have been found to disagree in the particular case.

When digestive disturbances are already present, the diet becomes necessarily restricted, and much depends then upon the nature of the indigestion, and sometimes also upon the tastes of patients as to the selection.

Milk, however, is the great staple in almost every emergency, and I am much hampered when it, too, cannot be taken in sufficient quantity. Fortunately this is rare, and I have had many patients who, although before trial protesting that it disagreed, have taken milk in the form of koumyss, peptonized, sterilized, or modified some other way, and have done well on it when other food could be given only sparingly or not at all; taking from two to four quarts of milk daily.

As long as we desire to increase the patient's weight, the hydrocarbons must be given liberally, and if possible in excess of the physiological standard; but good milk, bread, butter, the well-cooked cereals and vegetables, and, for those who like it, fat meat, are, as a rule, much more acceptable and attended by better results than fat emulsions, no matter how much their taste may be disguised.

The cooking and serving of food for consumptive patients is also of the greatest importance, and I fancy that often as much depends upon it as upon the particular article given.

In our direct or special treatment of pulmonary tuberculosis,

*Therapeutic Gazette*, October 15, 1890.

I confess I have little faith in drugs and their continuous administration. Some remedies, as we know, not interfering with the digestive organs, and which are not reducing agents to the blood, may possibly be employed, if we note improvement under their use.

I have for these reasons abstained from the internal use of mercurials, the iodides and creasote; and also the hypodermic injections of iodine and gold chlorides I have not been willing to apply, because of the known destructive effect of iodine upon the blood corpuscles, and because the injections are followed by fever, loss of appetite and *malise*, all of which are contrary to the principles of nutrition.

We cannot hope to cure a patient suffering from wasting disease by reducing him still further in applying remedies which, upon their face, act injuriously.

I have continued the use of tuberculin, first, because I have been able to so administer it as to utterly avoid fever, *malise*, or loss of appetite or any other unfavorable or disagreeable symptom, and because the patients have made remarkable local and general improvement under its use, and a gain in body weight has been observed in almost every case. Second, because from my study and investigation of its action when given in proper doses to suitable cases under certain precautions, I have come to the conclusion that its action upon tubercular tissue is selective, acting as a gentle stimulant or irritant to the periphery of the tubercular tissue, whereby the local nutrition is benefited, the encapsuling of the tubercle is favored and the advance of the disease arrested, through the increase of the circulation, whereby more blood reaches the affected part in which the artificial stimulation is induced. It is therefore an aid to the local nutrition of the affected part, but it is only one aid, and can never be depended upon to the exclusion of many other aids.

I believe that Liebreich's proposition of cantharidin as a remedy is based upon the same principle and has seemed beneficial in the few cases I have had occasion to employ it. Its stimulating and irritant action is not upon tubercular tissue as such, but upon all tissues the subject of present irritation and inflammation. I have, however, seen several abscesses from its use under every antiseptic precaution, and have exceptionally seen the injections followed by rigor and rise of temperature to 102. 5 deg. F., lasting several days, when two-tenths of a milligramme had been injected, which caused me to reduce the doses and would lead me to its abandonment if I should find such occurrences unavoidable.

Nothing can be expected of inhalations with the view of attacking the bacilli, simply because the germicides inhaled and the tubercle bacilli never come in contact. Not even in the mouth, pharynx and larynx have I seen any curative influences upon tubercular disease from such inhalations. The spray from soothing remedies, stimulants and astringents to the mucous membrane of the respiratory tract when inhaled does have a controlling influence upon the catarrhal processes.

The pneumatic cabinet has been a much-abused apparatus because it has been used, not for what it can do, but for what it was hoped to do, namely, to cure consumption.

Patients have been subjected to treatment with it because they had lung disease, instead of because this lung disease presented indications which the cabinet was capable of complying with.

Like many other means, it has not fulfilled our expectations because we have expected the impossible.

Its chief indication in pulmonary tuberculosis is for the production of a better circulation through the lung, which it certainly will accomplish if judiciously applied, and if that were all that is necessary, it would cure the disease.

The good influence upon the circulation is manifested upon the pulse for hours after its use, and also by the circumstantial evidence that in several hundred patients in whose cases I have made more or less use of it, we never had a single occurrence of hemorrhage or bloody expectoration during the periods of its employment, and among these cases were a number who had a history of frequent hemorrhage and bloody sputum. In several cases the present bloody expectoration ceased promptly upon its use.

The pneumatic cabinet is also useful for the accomplishment of a better ventilation of the affected lung, and for the recovery of collapsed air-cells and lobules, which would otherwise become atrophied and lost.

Its use is contra-indicated in all acute inflammatory processes and complications, and also where from increased lymphatic absorption, a distinct rise of temperature follows its application. It appears with its employment, as well as with all other useful means, that the influence it exerts is favorable to the local and general nutrition and the restoration or improvement of impaired functions.

As to hydropathic applications, the cold rub, with or without the addition of salt to the water, is certainly beneficial in not only accustoming the body to the more abrupt change of temperature, thereby diminishing or overcoming the liability to take cold; it is in addition a stimulant to the peripheral circulation and to the nervous system, and there are few cases of the disease where its systematic employment is not beneficial.

A bath towel is wrung comparatively dry out of cold water, reduced in temperature upon successive days until we reach 50 deg. F. or even less. The patient is then rapidly rubbed off and dried, the whole procedure requiring less than a minute, unless, if the reaction is not prompt, additional dry friction is necessary.

Such applications should be made regularly before dressing in the morning, and the patient can then take a little exercise out-of-doors before breakfast.

Only the weakest patients may take a hot drink preceding the rubbing, or return to bed for some rest after it.

Simple as this procedure is, it is nevertheless an important aid, and should not be neglected as unimportant just because it is so simple. I have abandoned the shower-baths as being more troublesome and inconvenient and also less agreeable to the patient, while not attended by greater benefits than the simple application spoken of. Systematic exercise, and in bed-patients the substitution of massage, is also highly essential for the best nutrition.

The exercise must, however, remain within the limits short of fatigue in all cases, and this applies to massage as well. Severer exertions, mountain-climbing, and such efforts as cause shortness of breath must be reserved for a period when the cure is completed, to be then used rather to clinch and to test the permanency of the result, instead of during the period of a cure. If we produce heart fatigue by the exercise, we interfere with, rather than aid, the nutritive processes, and the experience I have recorded elsewhere upon the detrimental effects of over-exertion,* to which I have frequently opportunity of adding, should convince any one that great care and circumspection is essential in strictly regulating both mental and physical exercise or labor for each individual patient according to his present condition.

In conclusion, I beg to urge upon the profession the great importance of early measures and the prompt employment of each and every means with which we can hope to aid recovery, and also my conviction that with the means we now possess we are not indifferently equipped for a successful encounter with the disease in its earlier stages.

* "The Detrimental Effects of Over-exertion on Pulmonary Phthisis," *Therap. Gaz.*, Dec. 15, 1890.

